# A Survey of Oral and Maxillofacial Biopsies Over a 23-year Period in the Southeast of Iran

**DOI:** 10.30476/DENTJODS.2021.90355.1487

**Published:** 2022-09

**Authors:** Mahsa Kalantari, Aida Alavi Samani

**Affiliations:** 1 Dept. of Oral and Maxillofacial Pathology, Oral and Dental Diseases Research Center, Kerman Dental School, Kerman University of Medical Sciences, Kerman, Iran; 2 Private Dentist, Student Research Committee, Kerman Dental School, Kerman University of Medical Sciences, Kerman, Iran

**Keywords:** Biopsy, Prevalence, Pathology, Oral, Iran

## Abstract

**Statement of the Problem:**

The prevalence of oral and maxillofacial lesions differs in various populations and is an important concern for health care providers.

**Purpose:**

The present study aimed to evaluate the relative frequency and distribution of oral and maxillofacial lesions in patients referred to the Department of Oral and Maxillofacial Pathology, Kerman Faculty of Dentistry in southeast Iran

**Materials and Method:**

In the present retrospective study, the collected data consisted of age, gender, lesion location, and the clinical and histopathological diagnoses of all the biopsy samples diagnosed in 23 years (1997–2020). The data were analyzed with SPSS 22, using the chi-squared test and ANOVA (*p*< 0.05).

**Results:**

From 2092 lesions with a definite diagnosis, 1202 (57.5%) and 890 (42.5%) cases belonged to female and male patients, respectively. The mean age of the subjects was 39.06±17.71 years, and the most frequent location of the lesions was the buccal mucosa (25.2%). The frequencies and number of non-neoplastic lesions, neoplasms, and premalignant lesions were 84% (n=1758), 13.3% (n=278), and 2.7% (n=56), respectively. Reactive lesions were the most common cases, with 34.6% (n=724), and lichen planus was the most frequent lesion with 18.1% (n=379). Squamous cell carcinoma was the most frequent lesion in patients >70 years of age (27.6 %).

**Conclusion:**

The present study provided useful data on the frequency and distribution of oral lesions over 23 years and made it possible to compare its results with those of studies carried out in other countries. Non-neoplastic lesions were the most common category, and lichen planus, pyogenic granuloma, and irritation fibroma were the most frequent lesions in descending order.

## Introduction

The oral and maxillofacial areas can be affected by a wide variety of lesions with different origins and characteristics [ 1 ]. Although it is sometimes possible to reach a clinical diagnosis based on clinical examinations, in the majority of cases, it is necessary to carry out further evaluations, including biopsies. Histopathological evaluation is the gold standard to reach a definitive diagnosis and render proper treatment to patients [ 2 - 4 ]. Given the wide range of oral and maxillofacial lesions, it is necessary for the oral healthcare specialists, including general dental practitioners, to have adequate knowledge on the clinical and demographic characteristics of these lesions because some of the benign lesions in this area might exhibit similar clinical or radiographic manifestations and might even resemble malignant lesions [ 5 - 7 ]. Unlike dental and periodontal lesions about which dentists have adequate knowledge, the diagnosis of some oral and maxillofacial lesions might pose a challenge. Therefore, collecting the data on biopsied lesions not only can increase knowledge about the prevalence and distribution of these lesions in the Iranian population but also can emphasize the lesions that oral healthcare specialists might encounter in their routine practice at a higher frequency [ 8 - 10 ].

Several studies have reported the relative prevalence of biopsied lesions of the oral and maxillofacial regions in different parts of the world. However, the majorities of these reports have been confined to a specific age group or a specific group of lesions, or have been based on clinical examinations without histopathological evaluations. Limited studies on histopathological diagnosis that included a full complement of oral lesions and patients in all age groups have been carried out on oral and maxillofacial lesions all over the world [ 11 - 22 ].

Considering the importance of knowledge about the epidemiologic properties of oral and maxillofacial lesions, and the early diagnosis and management of these lesions in each geographical location, the present study aimed to evaluate all the samples diagnosed in the Department of Oral and Maxillofacial Pathology of Kerman Faculty of Dentistry, in Kerman Province, as the largest province in Iran, over 23 years.

## Materials and Method

The present retrospective study was carried out on the biopsies taken from July 1997 to July 2020 from the patients referred to the Department of Oral and Maxillofacial Pathology, which had undergone histopathological evaluations. The Ethics Committee of Kerman University of Medical Sciences approved the protocol of the study under the code K.97.285.

The demographic data of the patients were collected from the database of the Department, which included age, gender, and other data on the location of the lesions, the clinical and histopathological diagnosis. The samples related to the repetition of the biopsies, which had already been diagnosed (e.g. for the excisional biopsy of a lesion that had already been diagnosed by using an incisional biopsy) were excluded. In addition, samples with descriptive and non-definitive diagnoses and normal tissues were excluded.

Based on the histopathological diagnosis, the lesions were categorized into three main groups of non-neoplas-tic, neoplastic, and premalignant lesions. Then the non-neoplastic lesions were divided into eight subgroups including reactive lesions, infectious and non-specific inflammatory lesions, cystic lesions (odontogenic or non- odontogenic), pigmented lesions, osseous lesions, salivary glands lesions, tooth-related and periodontium-related lesions, and immune system-related lesions. The neoplastic lesions were divided into two subgroups including benign (odontogenic and non-odontogenic) and malignant. The data were analyzed with SPSS 22, using the chi-squared test, ANOVA, and post hoc tests at a significance level of *p*< 0.05.

## Results

Of 2329 samples, 45 samples were excluded due to the observation of normal tissue in microscopic evaluations, and 192 were excluded due to indefinite diagnosis or repetition of the biopsy from a previous lesion. The final sample size consisted of 2092 biopsies, of which 1202 (57.5%) and 890 (42.5%) had been taken from female and male patients, respectively (with a 1.3:1 female-to-male ratio). The mean age of the patients was 39.06± 17.71 years, with an age range of 7 months to 105 years. The majority of the patients (18%) were in their fourth decade of life.

In terms of the classification of the histopathological diagnosis, 1758 cases (84%) were non-neoplastic lesions, 278 cases (13.3%) were neoplastic lesions, and 56 cases (2.7%) were premalignant lesions. The non-neoplastic lesions were more common in female subjects compared to neoplastic and premalignant lesions, and the difference was significant (*p*= 0.026). The mean age of the patients with premalignant lesions was higher than that of patients with neoplastic and non-neoplastic lesions; neoplastic lesions were detected in patients with a higher mean age compared to non-neoplastic lesions (*p*< 0.001).

The most common areas affected were the buccal mucosa (n=529), the mandible (n=265), and the maxillary gingiva (n=215). The other areas were the mandibular gingiva (n=206), the tongue (n=199), the maxilla (n=196), the labial mucosa (n=147), the vestibule (n=96), the palate (n=87), the alveolar ridge (n=69), the floor of the mouth (n=20), the alveolar socket (n=14), the skin (n=12), and the salivary glands (n=8). In 29 cases, the data were missing about the location of the lesion. The buccal mucosa was the most common area of involvement for the non-neoplastic and premalignant lesions; however, the tongue was affected by the neoplastic lesions at a significantly higher rate (*p*<0.05). Table 1 presents the distribution of the lesions in the three main groups in terms of gender, age, and the most common location of involvement.

**Table 1:** The frequencies of the lesions in three main groups in terms of age, mean age, and the most common location

Lesion	No. (%)	Gender		Mean age	The most common location
		Male	Female		
Neoplastic	278 (13.3%)	130 (6.2%)	148 (7.1%)	45.12±16.32	Tongue (19.6%)
Non-neoplastic	1758 (84%)	730 (34.9%)	1028 (49.1%)	37.85±17.12	Buccal mucosa (25.7%)
Premalignant	56 (2.7%)	30 (1.4%)	26 (1.3%)	49.51±12.77	Buccal mucosa (44.6%)

### Non-neoplastic lesions

The most prevalent subgroups of non-neoplastic lesions were reactive lesions (n=724), immune system-related lesions (n=433), cysts (n=284) (including 271 odontogenic and 13 non-odontogenic cyst), tooth- and periodontal tissue-related lesions (n=93), infectious and non-specific inflammatory lesions (n=72), osseous lesions (n=53), salivary glands lesions (n=5), and pigmented lesions (n=48). Lichen planus (n=379), pyogenic granuloma (n=212), and irritation fibroma (n=201) were the most prevent non-neoplastic lesions in descending order. Table 2 presents the 5 most prevalent lesions in each subgroup of non-neoplastic lesions in terms of gender, age, and the most common area(s) affected.

**Table 2:** The gender and age distribution and the most common locations of involvement of non-neoplastic lesions (five most prevalent lesions in each subgroup)

Lesion	No. (%)	Gender		Mean age	Age range	The most common location
		Male	Female			
Reactive lesions						
Pyogenic granuloma	212 (29.3%)	71	141	33.46±15.66	6-70	Maxillary gingiva (34%)
Irritation fibroma	201 (27.8%)	77	124	38.69±15.43	6-77	Buccal mucosa (37.3%)
Peripheral ossifying fibroma	83 (11.3%)	38	45	26.65±11.69	4-59	Maxillary gingiva (54.2%)
Hyperkeratosis	78 (10.8%)	51	27	47.62±14.39	4-81	Buccal mucosa (55.1%)
Epulis fissuratum	71 (9.8%)	24	47	58.76±14.23	6-105	Vestibule (97.2%)
Immune-mediated lesions						
Lichen planus	379(87.5%)	135	244	44.99±13.91	10-81	Buccal mucosa (71.5%)
Pemphigus vulgaris	39 (9%)	16	23	36.87±11.28	31-64	Buccal mucosa (35.9%)
Cicatricial pemphigoid	11 (2.5%)	5	6	46.50±6.73	24-78	Mandibular gingiva (45.5%)
Erythema multiform	3 (0.7%)	2	1	26.66±13.57	14-41	Labial mucosa (33.3%)
Lupus erythematosus	1 (0.2%)	.0	1	43	-	Buccal mucosa (100%)
Odontogenic cysts						
Radicular cyst	160(58.9%)	88	72	31.44±15.60	6-75	Mandible (53.1%)
Dentigerous cyst	59 (21.9%)	25	34	18.05±14.54	2-58	Mandible (59.3%)
Odontogenic keratocyst	31 (11.5%)	16	15	32.35±12.83	16-71	Mandible (71%)
Unicystic ameloblastoma	7 (2.6%)	1	6	32.43±13.80	16-55	Mandible (100%)
Calcifying odontogenic cyst	6 (2.2%)	4	2	34.01±24.54	12-75	Maxilla (83.3%)
Tooth- and periodontal-related lesions						
Periapical granuloma	72 (78%)	29	43	33.44±12.95	14-63	Maxilla (55.6%)
Periodontitis	8 (8.8%)	4	4	41.42±10.37	30-58	Mandibular gingiva (62.5%)
Dental follicle	7 (6.6%)	2	5	27.75±10.68	15-38	Mandible (85.7%)
Gingival fibromatosis	4 (4.4%)	1	3	15.25±7.13	8-25	Maxillary gingiva (50%)
Plasma cell gingivitis	1 (1.1%)	.0	1	20	-	Mandibular gingiva (100%)
Inflammatory and infectious lesions						
Nonspecific ulcer	26 (36.1%)	7	19	49.01±18.43	14-79	Tongue (26.9%)
Chronic inflammation	26 (36.1%)	11	15	35.02±13.67	13-60	Maxillary gingiva (26.9%)
Granulation tissue	7 (9.7%)	3	4	33.60±19.15	13-65	Maxillary gingiva (28.6%)
Granulomatous lesions	3 (4.2%)	2	1	32.33±17.38	19-52	Labial mucosa (100%)
Aphthous	3 (4.2%)	3	.0	24.33±11.59	11-32	Buccal mucosa (66.7%)
Non-odontogenic cysts						
Nasopalatine duct cyst	6 (54.5%)	2	4	50.12±14.17	31-70	Maxilla (83.31%)
Epidermoid cyst	3 (27.3%)	1	2	30.66±7.09	23-37	Skin (100%)
Lymphoepithelial cyst	2 (18.21%)	1	1	43.50±26.16	25-63	Tongue (50%)
Pigmented lesions						
Melanotic macula	23 (47.9%)	4	19	42.43±14.79	20-87	Labial mucosa (26.1%)
Amalgam tattoo	13 (27.1%)	6	7	40.15±13.83	21-58	Alveolar ridge (30.8%)
Exogenic pigmentation	8 (16.7%)	2	6	46.12±15.85	19-68	Buccal mucosa (37.5%)
Focal melanosis	2 (4.2%)	.0	2	46.50±13.43	37-56	Buccal mucosa (100%)
Blue nevus	2 (4.2%)	1	1	40.50±40.30	12-69	Palate (100%)
Osseous lesions						
Central giant cell granuloma	26 (49.1%)	9	17	28.04±19.01	1-67	Mandible (57.7%)
Osteomyelitis	10 (18.9%)	6	4	32.31±17.56	6-55	Mandible (80%)
Cemento-osseous dysplasia	6 (11.3%)	1	5	34.83±13.52	22-50	Mandible (83.3%)
Traumatic bone cyst	4 (7.5%)	1	3	34.50±13.52	22-51	Mandible (100%)
Fibrous dysplasia	4 (7.5%)	2	2	20.01±9.55	10-33	Maxilla (50%) and Mandible (50%)
Salivary gland lesions						
Mucocele	37 (69.8%)	20	17	21.65±10.34	4-42	Labial mucosa (83.8%)
Sialoadenitis	6 (11.3%)	2	4	39.83±10.53	29-54	Salivary glands (66.7%)
Sjögren syndrome	4 (7.5%)	2	2	52.33±11.29	40-62	Salivary glands (75%)
Salivary duct cyst	2 (3.8%)	2	.0	45.50±24.78	28-63	Labial mucosa (50%) and Palate (50%)
Cheilitis glandularis	2 (3.8%)	.0	2	47.50±30.41	26-69	Labial mucosa (100%)

### Neoplastic lesions

Of 278 neoplastic cases in the present study, 166 cases (59.7%) were benign, and 112 (40.3%) were malignant. There were no significant differences in the distribution of benign and malignant neoplasms between the male and female patients (*p*= 0.6). However, benign odontogenic neoplasms were significantly more prevalent in female patients compared to male patients (*p*= 0.05).

The majority of benign neoplasms (n=138) were non-odontogenic, and a minority of them (n=28) were odontogenic. Of all the non-odontogenic benign tumors, squamous papilloma (n=42) and acquired melanocytic nevus (n=17) were the most frequent lesions; of all the benign odontogenic tumors, ameloblastoma (n=11) and odontoma (n=9) were the most common ones.

Of 112 malignant neoplasms evaluated in the present study, squamous cell carcinoma (SCC) was the most prevalent one with 82 cases, and odontogenic carcinosarcoma, olfactory neuroblastoma, and plasmacytoma were the least frequent ones with one case each. Table 3 presents the gender and age distributions and the most common location of involvement for the five most common lesions in each subgroup of neoplastic lesions.

**Table 3:** The gender and age distribution and the most common locations of involvement of neoplastic lesions (five most prevalent lesions in each subgroup)

Lesion	No. (%)	Gender		Mean age	Age range	The most common location
		Male	Female			
Benign non-odontogenic						
Squamous papilloma	42 (30.4%)	24	18	35.60±18.92	5-70	Palate (28.6%)
Acquired melanocytic nevus	17 (12.3%)	6	11	33.80±14.41	16-59	Buccal mucosa (23.5%)
Lipoma	16 (11.6%)	7	9	51.06±15.01	7-72	Buccal mucosa (62.5%)
Giant cell fibroma	14 (10.1%)	6	8	38.70±22.14	2-76	Buccal mucosa (42.9%)
Pleomorphic adenoma	10 (7.2%)	5	5	8.75±16.92	25-67	Palate (70%)
Benign odontogenic						
Ameloblastoma	11 (39.2%)	3	8	45.20±15.28	27-72	Mandible (72.7%)
Odontoma	9 (32.1%)	2	7	25.66±24.42	2-61	Maxilla (55.6%)
Odontogenic myxoma	4 (14.2%)	1	3	26.50±3.69	23-31	Maxilla (50%) and Mandible (50%)
Central odontogenic fibroma	2 (7.1%)	2	.0	26.87±19.01	16-54	Mandible (100%)
Adenomatoid odontogenic tumor	2 (7.1%)	1	1	25	-	Maxilla (50%) and Mandible (50%)
Malignant						
Squamous cell carcinoma	82 (73.2%)	38	44	58.50±16.19	15-92	Tongue (39%)
Verrucous carcinoma	5 (4.5%)	3	2	66.60±23.58	35-88	Vestibule (60%)
Adenoid cystic carcinoma	4 (3.6%)	.0	4	49.75±23.78	32-83	Palate (66.7%)
Osteosarcoma	4 (3.6%)	.0	4	20.77±5.73	16-29	Maxilla (50%) & mandible (50%)
Mucoepidermoid carcinoma	3 (2.7%)	2	1	57.06±4.61	16-83	Palate (66.7%)

### Premalignant lesions

Of 56 premalignant lesions evaluated in the present study, 55 cases were dysplastic epithelium related to leukoplakia or erythroplakia, and one case was actinic cheilitis. The most frequent location of involvement for premalignant lesions was the buccal mucosa, with 25 cases (44.6%) (Table 4).

**Table 4:** The gender and age distribution and the most common locations of involvement of premalignant lesions

Lesion	No. (%)	Gender		Mean age	Age range	The most common location
		Male	Female			
Dysplastic epithelium	55 (98.2%)	30	25	49.50±12.89	18-83	Buccal mucosa (45.5%)
Actinic cheilitis	1 (1.8%)	0	1	50	-	Labial mucosa (100%)

### Ten most common lesions in terms of age and gender

**Figure 1:**
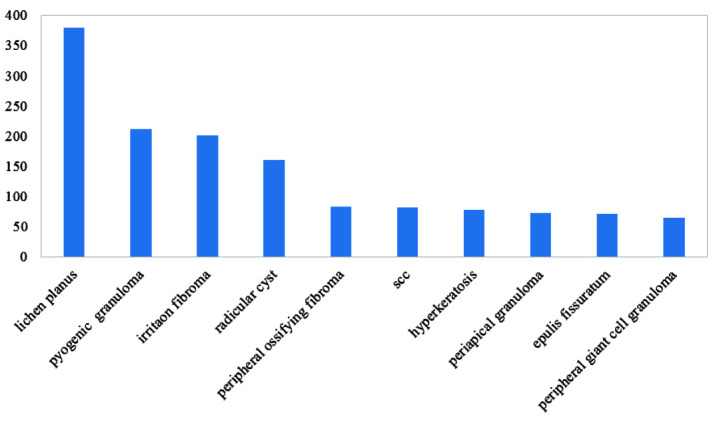
The relative frequencies and distribution of the 10 most common lesions in histological evaluations

**Figure 2:**
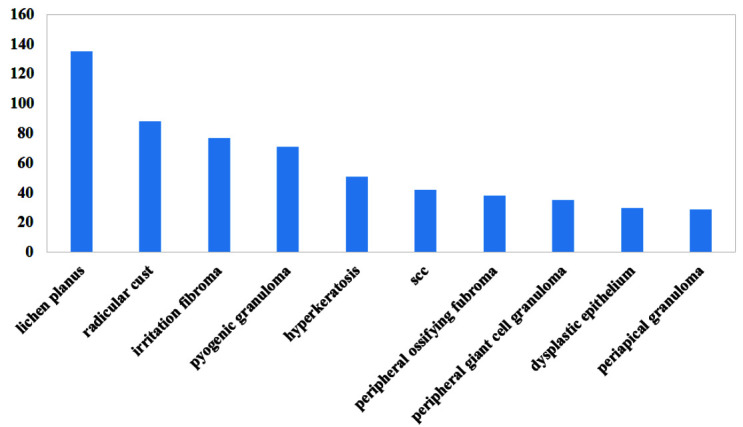
The relative frequencies and distribution of the 10 most common lesions in histological evaluations in male patients

**Figure 3:**
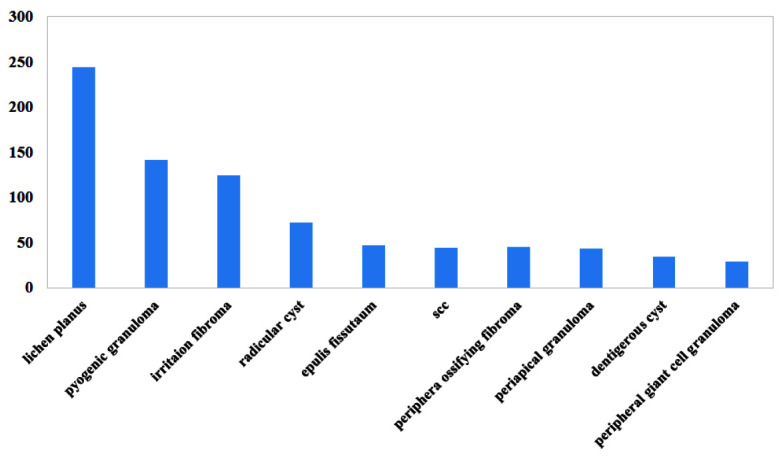
The relative frequencies and distribution of the 10 most common lesions in histological evaluations in female patients

A wide range of lesions affecting the oral and maxillofacial region was observed in the present study (106 different lesions). The most commonly reported lesions in the histopathological evaluations were lichen planus (18.1%), pyogenic granuloma (10.1%) and irritation fibroma (9.6%). Figures 1, 2 and 3 present the frequencies of 10 most common lesions in microscopic evaluations separately in male and female patients. Evaluation of the most common lesions in terms of age decades showed that the dentigerous cyst and pyogenic granuloma were the most common lesions in the first and second decades of life, respectively. The most frequent lesions in the third to the seventh decades of life and the eighth and ninth decades of life were lichen planus and SCC, respectively. In addition, in 1224 cases (58.5%), there was an agreement between the clinical and histopathological evaluations; the agreement in the case of mucocele, lichen planus, and epulis fissuratum was higher compared to other lesions with 89.2%, 88.9%, and 88.7%, respectively.

## Discussion

Studies on the frequency of oral and maxillofacial lesions are an essential aspect of oral healthcare programs and provide epidemiologic data on the distribution of these lesions in different populations [ 23 - 24 ]. In the present retrospective study, 2092 oral and maxillofacial biopsies were evaluated. Evaluation of the data showed a higher frequency of these lesions in female subjects compared to male subjects, consistent with other reports [ 7 - 10 , 12 ]. Evaluation of the number of biopsies in terms of the different decades of life showed that the majority of the lesions had been diagnosed in the fourth decade of life, consistent with the results of studies in Ghana [ 13 ] and Jordan [ 7 ]. In the present study, reactive lesions were reported as the most prevalent subgroup (31.1%), in line with the majority of previous studies [ 7 , 10 , 12 , 14 ]. Since the oral cavity is subject to various traumatic and irritating agents, such a finding is expected [ 25 - 26 ]. In this group, pyogenic granuloma was the most common lesion (9.1% of all the lesions). Although some studies have reported a similar finding [ 19 , 14 ], the majority of studies have reported irritation fibroma as the most common reactive lesion [ 6 - 7 , 11 - 12 ]. In addition, pyogenic granuloma had the highest prevalence in female patients and the maxillary gingiva, in agreement with the results of previous studies [ 10 , 14 ].

Immune system-related lesions were the second most prevalent subgroup in the present study (18.6%). Lichen planus was the most common lesion in this subgroup and the most common lesion of all the lesions. In line with the results of studies in the United Kingdom [ 6 ] and Kuwait [ 11 ], lichen planus was more common in female patients and the buccal mucosa. Evaluation of similar articles shows significant variations in the arrangement of common subgroups.

In a study by Monterio *et al.* [ 12 ], cystic lesions constituted the second most common subgroup of lesions; in studies by Ali *et al.* [ 11 ] and in a study by Alkhateeb *et al.* [ 7 ], inflammatory lesions, and in a study by Jones and Franklin [ 6 ], tooth-related lesions were the second most common subgroups. It appears that one of the reasons for these differences is the use of different classification systems in different studies. A search in the literature did not show any two studies that have used a similar classification system for lesions. In addition, ethnic and geographical variations, differences in sample sizes, and the mechanism of referrals of the patients might cause these differences [ 27 ].

In the present study, lichen planus was the most frequent lesion; however, none of the similar studies has reported such a finding. Pyogenic granuloma [ 10 , 14 ], irritation fibroma [ 6 - 7 , 11 - 12 ], and radicular cyst [ 28 ] have been reported as the most frequent lesions in other studies; only a study by Hatem *et al.* [ 10 ] reported lichen planus as the second most common lesion. It should be noted that the therapeutic centers send the samples to the oral pathology centers, and these treatment centers are significantly different from each other. Since patients with various mucosal lesions from southeast Iran are referred to the Department of Oral Medicine, Faculty of Dentistry, Kerman University of Medical Sciences, a considerable proportion of the biopsies received in this department are mucosal lesions; therefore, the samples are significantly different from the samples in an oral pathology center in a hospital, where the samples comprise a higher proportion of neoplastic lesions in an oral pathology center in a hospital, where the samples comprise a higher proportion of neoplastic lesions. Akinmoladum *et al.* [ 26 ] evaluated the samples in the oral pathology department of a hospital and reported that 51.3% of the cases were benign neoplasms, and 29.6% and 19.1% were malignant neoplasms and reactive lesions, respectively, confirming what was discussed above.

Cystic lesions were the third most common subgroup (12.2%) in the present study. In accord with the majority of previous studies, radicular and dentigerous cysts were the most frequent cystic lesions, in descending order, with a higher frequency in the mandible [ 6 , 10 , 12 , 27 ]. Of all the non-odontogenic cysts, the nasopalatine duct cyst exhibited a higher frequency, consistent with the results of studies in Jordan [ 7 ] and Portugal [ 12 ].

In the present study, neoplastic lesions (11.9%) were less frequent than the non-neoplastic lesions. The prevalence of these lesions has been reported in other studies at 9.5% [ 12 ] and 27.6% [ 27 ] in Brazil, and 14.7% in Kuwait [ 11 ]. In the present study, benign neoplasms were more frequent than the malignant lesions, compatible with the results of other studies [ 10 , 14 , 26 - 27 ]. Squamous papilloma (1.8% of all the lesions) was the most frequent benign neoplasms in the present study. Jones and Franklin [ 6 ] and Ali *et al.* [ 11 ] reported similar results; however, Alkhateeb *et al.* [ 7 ] and Monterio *et al.* [ 12 ] reported that squamous papilloma was the second most common benign neoplasm after pleomorphic adenoma. Benign odontogenic tumors comprised 1.1% of all the samples in the present study. The prevalence of these lesions was 2.3% in Kuwait [ 11 ] and 3.4% in Lybia [ 10 ], which confirms a low prevalence of these lesions. In the present study, similar to in the results yielded by the studies of Franklin and Jones [ 6 ] and Hatem *et al.* [ 10 ], ameloblastoma was the most prevalent odontogenic tumor, with a higher prevalence in the mandible.

In the present study, malignant neoplasms comprised 4.8% of all the lesions. The prevalence of malignant neoplasms in other studies has been different, with 1.9% in a study by Mendez *et al.* [ 27 ], 7.6% in a study by Ali *et al.* [ 11 ] and 15% in a study by Monterio *et al.* [ 12 ]. The most frequent malignancy in the present study was SCC, with 3.5% of all the lesions, consistent with other studies. The most common location affected by SCC was the tongue, with a mean age of 58.5 years of the patients, in line with previous studies [ 6 , 10 - 12 , 26 - 27 ]. Of all the malignant salivary gland neoplasms, adenoid cystic carcinoma and mucoepidermoid carcinoma exhibited the highest prevalence, in agreement with the results of studies in Brazil [ 9 ] and Portugal [ 12 ].

In the present study, neoplastic lesions (11.9%) were less frequent than the non-neoplastic lesions. The prevalence of these lesions has been reported in other studies at 9.5% [ 12 ] and 27.6% [ 27 ]. in Brazil, and 14.7% in Kuwait [ 11 ]. In the present study, benign neoplasms were more frequent than the malignant lesions, compatible with the results of other studies [ 10 , 14 , 26 - 27 ]. Squamous papilloma (1.8% of all the lesions) was the most frequent benign neoplasms in the present study. Jones and Franklin [ 6 ] and Ali *et al.* [ 11 ] reported similar results; however, Alkhateeb *et al.* [ 7 ] and Monterio *et al.* [ 12 ] reported that squamous papilloma was the second most common benign neoplasm after pleomorphic adenoma. Benign odontogenic tumors comprised 1.1% of all the samples in the present study. The prevalence of these lesions was 2.3% in Kuwait [ 11 ] and 3.4% in Lybia [ 10 ], which confirms a low prevalence of these lesions. In the present study, similar to in the results yielded by the studies of Franklin and Jones [ 6 ] and Hatem *et al.* [ 10 ], ameloblastoma was the most prevalent odontogenic tumor, with a higher prevalence in the mandible.

## Conclusion

Generally, the majority of the lesions in the present study were non-neoplastic, and lichen planus was the most common lesion among all the lesions. Malignant neoplasms comprised 4.8% of all the lesions, and SCC was the most frequent malignancy (73.2%). The present study not only did provide useful information about the frequency and distribution of oral lesions and assistance for the proper differential diagnosis of these lesions but also carried out a comparison of this prevalence with the data available from other countries.

## Acknowledgment

The authors would like to thank Research Committee of Kerman Medical University for their financial support.

This study was approved by the Ethics Committee of Kerman University of Medical Sciences with the code of K.97.285.

## Conflict of Interest

The authors declare that they have no conflict of interest.
